# High altitude population of *Arabidopsis thaliana* is more plastic and adaptive under common garden than controlled condition

**DOI:** 10.1186/s12898-017-0149-5

**Published:** 2017-12-13

**Authors:** Akanksha Singh, Sribash Roy

**Affiliations:** 1 Genetics and Molecular Biology Division, CSIR-National Botanical Research Institute, Lucknow, Uttar Pradesh 226001 India; 2 Academy of Scientific and Innovative Research (AcSIR), Anusandhan Bhawan, 2 Rafi Marg, New Delhi, 110 001 India

**Keywords:** West Himalaya, *Arabidopsis thaliana*, Phenotypic differentiation, Phenotypic plasticity, Common garden, Controlled condition, Selection

## Abstract

**Background:**

Population differentiation and their adaptation to a particular environment depend on their ability to respond to a new environment. This, in turn is governed to an extent, by the degree of phenotypic plasticity exhibited by the populations. The populations of same species inhabiting different climatic conditions may differ in their phenotypic plasticity. Himalayan populations of *Arabidopsis thaliana* originating from a steep altitude are exposed to different climatic conditions ranging from sub-tropical to temperate. Thus they might have experienced different selection pressures during evolution and may respond differently under common environmental condition.

**Results:**

Phenotypic plasticity and differentiation of natural populations of *A. thaliana* grown under common garden and controlled conditions were determined. A total of seventeen morphological traits, their plasticity, association between traits and environment were performed using 45 accessions from three populations. Plants from different altitudes differed in phenotypes, their selection and fitness under two conditions. Under both the conditions lower altitude population was characterized by higher leaf count and larger silique than higher and middle altitude population. Flowering time of high altitude population increased while that of low and medium altitude decreased under controlled condition compared to open field. An increase in seed weight and germination was observed for all the population under open field than controlled. Rosette area was under divergent selection in both the condition. The heritability of lower altitude population was the highest under both the conditions, where as it was the least for higher altitude population further indicating that the high altitude populations are more responsive towards phenotypic changes under new environmental conditions. Ninety-nine percent of variability in traits and their plasticity co-varied with the altitude of their origin. The population of high altitude was more plastic and differentiated as compared to the lower altitude one.

**Conclusions:**

*Arabidopsis thaliana* population native to different altitudes of the west Himalaya responds differently when grown under common environments. The success of high altitude population is more in common garden than the controlled conditions. The significant variability in phenotype and its association with altitude of origin predicts for non-random genetic differentiation among the populations.

**Electronic supplementary material:**

The online version of this article (10.1186/s12898-017-0149-5) contains supplementary material, which is available to authorized users.

## Background

Plants being sessile organisms are frequently exposed to heterogeneous environmental conditions. The variation in environmental condition influences the evolution of traits and their differentiation. It can favour either local adaptation (adaptive differentiation/specialization) or evolution of plastic genotypes that respond through phenotypic plasticity (generalization). Both adaptive differentiation and phenotypic plasticity acts as major factor in generation of inter-individual variation and plant diversity. The amount of variation both within and among populations influences the response towards the changing environment. There has been a long standing interest to understand the role of plasticity on the performance of individuals, populations and species in new environment [1]. Variations in the environmental conditions can lead to both phenotypic and genotypic variations among the populations over a long period of time. Natural selection acting on phenotypes and their plasticity can cause evolution of populations leading to local adaptation.

*Arabidopsis thaliana* (L.) Heynh., has recently been studied as a model plant species for the ecological and eco-genomics studies [2, 3]. This is mainly because of its ease of growth and ubiquitous presence as well as publicly available large information resources. Being a model organism, it is important to quantify the diversity of this plant contributing to adaptation throughout its geographical distribution. There are reports of environmental influence on phenotypic traits like number of fruits, germination, length and width of fruits, flowering time, flooding response, etc. using field grown *Arabidopsis* plants [4–7]. Similarly, a few studies investigated the fitness of *Arabidopsis* mutants grown under field conditions and commented on morphological changes that are likely to result from varied environmental influence [3, 4, 8]. There are also reports on phenotypic, physiological and gene expression profiling of plants grown either in simulated or controlled condition [9–12]. Phylogeography of *A. thaliana* in western Eurasia [13, 14] and its rapid expansion throughout the world has also been studied [15]. Though, a lot of studies have been conducted throughout the world including other European populations [6, 11, 13, 14, 16–19], no such detailed studies have been conducted on highly diverse populations from West Himalaya. This incomplete sampling could limit our knowledge on extent of variation known in *A. thaliana*.

The West Himalayan populations are inhabited along wide altitude ranging from ~ 700 to ~ 3400 m amsl, which is also the altitudinal maxima of *A. thaliana* distribution reported so far. In contrast to predominantly temperate climatic condition in other European and American countries, *A. thaliana* growing along Himalayan altitude are exposed to sub-tropical to temperate climatic conditions [20]. Thus historically, these populations might have experienced different evolutionary pressure than the other world populations. However, these populations were never described earlier in terms of their phenotypic response towards different environmental conditions. These populations provide an opportunity for studying the effect of different environmental factors and their combinations on phenotypic diversity and differentiation. The strong variation in environmental factors along the steep altitude [17, 21] can impose strong selection on traits leading to their adaptation [22]. The highest altitude population (~ 3400 m amsl) which is exposed to extreme weather conditions might have evolved differently as compared to the more benign lower altitude population. Thus the fitness of this population may be higher under a common but heterogeneous environment as compared to lower altitude population. These populations are relatively old and harbour significant amount of genetic variations between the populations [23, 24]. Interestingly, these populations were also found to be genetically quite different from the rest of the world populations [23].

Here, we measured the phenotypic differentiation and plasticity of natural populations of *A. thaliana* from West Himalaya. A few studies had shown and argued that in order to understand population differentiation a relative analysis of the field and controlled grown plants is essential [5, 25, 26]. A significant variation in fitness has also been observed in mutants grown in the two conditions [26]. We compared the level of plasticity of these populations exhibited due to interaction between genotypes and environment. Specifically, we asked the following questions: Do the populations from different altitudes differ in the expression of traits in the common garden and controlled conditions (i.e. plasticity)? Are these differentiations population specific in either conditions, and related to altitude? How the populations differ in the selection of the traits in the two conditions? Under which of the two novel conditions plants show more fitness? The variations in phenotype could be either due to adaptive differentiation or random by genetic drift. An association of phenotype under common growth condition to its native altitude and climate predicts for an adaptive genetic differentiation of these populations reducing chance of being a random processes [17]. These differences are also expected to indicate the abiotic stress experienced by the populations in the field conditions.

## Results

### Trait variation among populations in either condition

The three populations grown in common garden (CG) and controlled condition (GH) varied significantly for all the vegetative and reproductive traits, except a few (Table 1). Besides being significantly variable, some of the traits also followed a trend of either increase or decrease with altitude. For example, in CG the lower altitude population (Deh) was characterized by lower leaf area as compared to middle (Mun) and higher (Chit) altitude population. Whereas, in the GH Deh was characterized by wider leaves, longer petioles and larger leaf area as compared to other two populations. Leaf counts and silique length were higher in Deh as compared to Mun and Chit in both the conditions. Chit flowered the most late, followed by Mun and the Deh in both the conditions. The post hoc Bonferroni pair wise comparison between the populations indicated that the lower (Deh) and the highest altitude populations (Chit) were most differentiated under both the conditions. However, the middle altitude population (Mun) showed a variable trend, being more similar to the Deh and Chit under GH and CG condition, respectively (Additional file 1: Table S1).

**Table 1:** Analysis of phenotypic variations under the two condition

	Degree of freedom	Common garden (CG)		Controlled condition (GH)	
		F value	P value	F value	P value
Vegetative traits (measured after 5 weeks of germination)					
Leaf number	2,42	6.8	*0.003*	88.292	*<* *0.0001*
Average leaf length (in cm)	2,42	42.043	*<* *0.0001*	16.796	*<* *0.0001*
Average leaf width (in cm)	2,42	119.872	*<* *0.0001*	130.668	*<* *0.0001*
Leaf area (leaf length * leaf width)	2,42	175.705	*<* *0.0001*	13.71	*<* *0.0001*
Leaf shape (leaf length/leaf width)	2,42	2.273	0.116	27.386	*<* *0.0001*
Petiole length (in cm)	2,42	77.393	*<* *0.0001*	5.187	*0.01*
Rosette area (rosette major axis * minor axis)	2,42	307.52	*<* *0.0001*	17.872	*<* *0.0001*
Rosette shape (rosette major axis/rosette minor axis)	2,42	1.458	0.244	3.775	*0.031*
Flowering time (estimated as number of days from bolting to germination)	2,42	13.406	*<* *0.0001*	155.423	*<* *0.0001*
Reproductive traits (measured after completion of life cycle)					
Plant height (in cm)	2,42	202.839	*<* *0.0001*	143.112	*<* *0.0001*
Inflorescence length (in cm)	2,42	80.379	*<* *0.0001*	98.212	*<* *0.0001*
Average silique length (in cm)	2,42	14.414	*<* *0.0001*	26.655	*<* *0.0001*
Average length of pedicel of silique (in cm)	2,42	17.827	*<* *0.0001*	0.852	0.434
Average distance between two siliques (in cm)	2,42	180.659	*<* *0.0001*	25.14	*<* *0.0001*
Number of flowers produced	2,42	23.672	*<* *0.0001*	3.289	*0.047*
Number of fruits	2,42	14.027	*<* *0.0001*	0.385	0.683
Fertility percentage (estimated as number of fruits/number of flowers * 100)	2,42	8.727	*<* *0.001*	23.922	*<* *0.0001*

ANOVA with phenotypic traits was performed to find the significance of trait differentiations in the three populations of *Arabidopsis thaliana* grown in the two environmental conditions independently. Significant trait differentiation values are in italics (P < 0.05)

### Trait variations between the two conditions

**Fig. 1:**
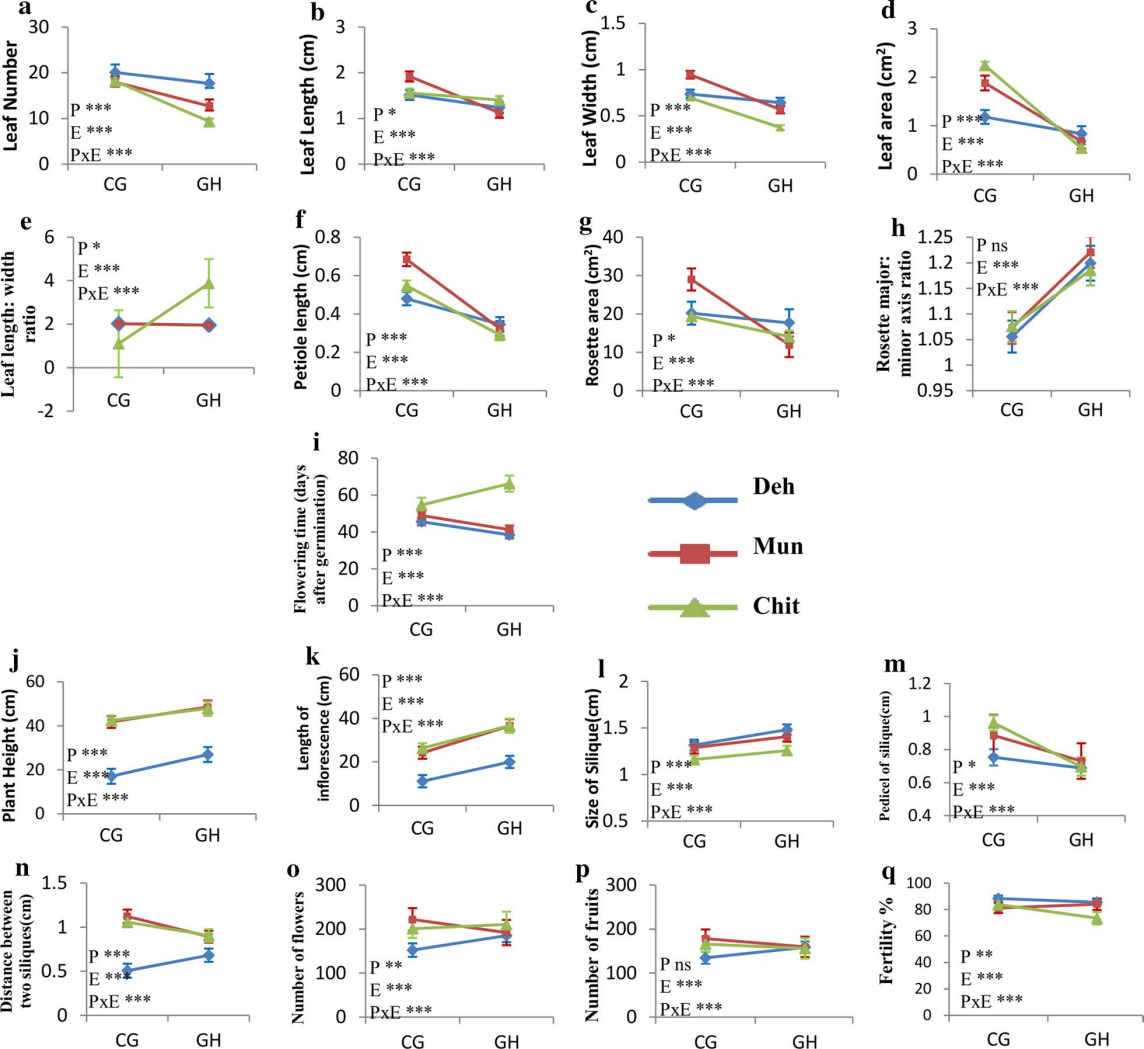
Graphical representation of difference in trait means of three populations in the two conditions. (**a**–**h**) Vegetative traits; (**i**) flowering time; (**j**-**q**) reproductive traits. Each point represents the least square mean of each population in common garden (CG) and controlled condition (GH). Bars above the lines represent the 95% confidence interval error bars. The results of ANOVA for each independent variable (P = population; E = growth environment) and their interaction (P × E) is also shown in the graph. *P < 0.05, **P < 0.01, ***P < 0.001, *ns* not significant (for **e** only two lines are displayed as values for Deh and Mun overlapped completely)

The differences in phenotypic trait expression between the two conditions varied at different level (Fig. 1; Additional file 1: Table S2). All the vegetative traits showed a significant decrease in GH as compared to CG for all the populations, except leaf length:width ratio and rosette major:minor axis ratio. The leaf length:width ratio (leaf shape) did not varied significantly for Deh and Mun in the two conditions. However, the leaf length:width ratio increased for Chit in GH. Thus the leaf was more rounded in Chit under CG as compared to GH condition. An increase in rosette major:minor axis ratio (rosette shape) was observed for all the populations in GH as compared to CG. Flowering time, expressed as days to bolting increased in Chit while that of Deh and Mun decreased in GH as compared to CG.

**Fig. 2:**
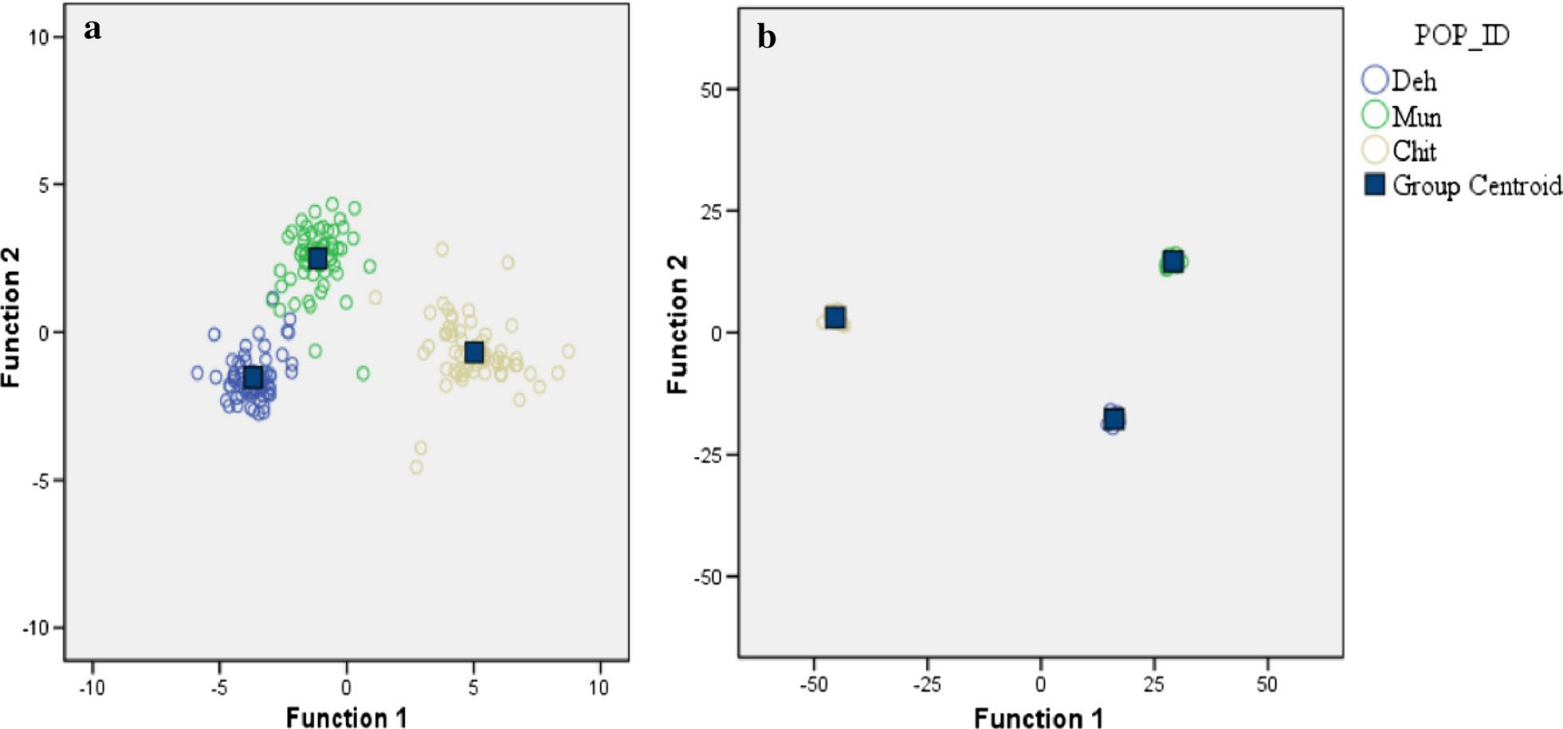
Clustering of the three populations on Discriminant function1 and Discriminant function 2 on the basis of: **a** phenotypic traits. **b** Amount of phenotypic plasticity

Further, among the reproductive traits, there was a decrease in plant height, inflorescence length and silique length in CG as compared to GH in all the populations. The length of pedicel of silique increased in CG as compared to GH in all the populations. A decrease in distance between two siliques was observed in lower altitude population (Deh) while it increased for Mun and Chit in CG as compared to GH. The total number of flowers produced decreased for Deh and Chit while it increased for Mun in CG. The number of fruits produced also differed significantly among the populations grown in the two conditions. Chit and Mun produced more number of fruits in CG as compared to GH where as Deh produced less fruits in CG as compared to GH. An increase in seed weight was observed for all the population in CG [population (P), P = 0.261; environment (E), P = 0.028; P × E, P = 0.049]. Germination percentage was significantly higher in CG produced seeds than GH [population (P), P = 0.233; environment (E), P < 0.0001; P × E, P = 0.183] in all populations. Overall, in either condition the populations had sufficient amount of phenotypic variations to distinguish the three populations as indicated by the discriminant analysis (Fig. 2a).

### Correlation between traits within and between two conditions

The expression of traits was generally similar in the two conditions except for leaf length and rosette area (Table 2). The leaf area was found to be negatively correlated between the two conditions (r = − 0.335, P = 0.025). However the correlation between the phenotypic traits within the two conditions differed both in the strength and direction (Additional file 1: Table S3). Among the vegetative traits, leaf number was negatively correlated with leaf area in CG, while it was positively correlated with leaf width, leaf area, petiole length, rosette area and negatively with leaf shape. Leaf shape showed a negative correlation with leaf area and positive with leaf length and width in CG, while in GH it was positively correlated with leaf length and negatively with leaf number, leaf width and rosette shape. Rosette shape was not correlated with any of the vegetative traits in CG, while it was negatively correlated with leaf length and leaf shape in the GH. Similar variation in both strength and direction of correlation was also observed for the reproductive traits (Additional file 1: Table S3). Most of the reproductive traits were positively correlated in the CG and negatively correlated in GH. The fruits produced in CG were positively correlated with most of the traits except (leaf number; r = − 0.375, P = 0.011) while in GH, it was correlated only to the number of flowers (r = 0.812, P < 0.001).

**Table 2:** Correlation of traits expressed between the two conditions

	Pearson correlation	P value
Vegetative traits (measured after 5 weeks of germination)		
Leaf number	*0.776*	< 0.0001
Average leaf length (in cm)	0.002	0.989
Average leaf width (in cm)	*0.485*	0.001
Leaf area (leaf length * leaf width)	*−* *0.335*	0.025
Leaf shape (leaf length/leaf width)	*0.782*	< 0.0001
Petiole length (in cm)	*0.323*	0.030
Rosette area (rosette major axis * minor axis)	0.018	0.906
Rosette shape (rosette major axis/rosette minor axis)	*0.896*	< 0.0001
Flowering time (estimated as number of days from bolting to germination)	*0.832*	< 0.0001
Reproductive traits (measured after completion of life cycle)		
Plant height (in cm)	*0.999*	< 0.0001
Inflorescence length (in cm)	*0.992*	< 0.0001
Average silique length (in cm)	*0.984*	< 0.0001
Average length of pedicel of silique (in cm)	*0.741*	< 0.0001
Average distance between two siliques (in cm)	*0.912*	< 0.0001
Number of flowers produced	*0.698*	< 0.0001
Number of fruits	*0.683*	< 0.0001
Fertility percentage (estimated as number of fruits/number of flowers * 100)	*0.712*	< 0.0001

Pearson correlation of all 17 morphological traits expressed between the two growth condition (CG and GH). Significant values are in italics

**Fig. 3:**
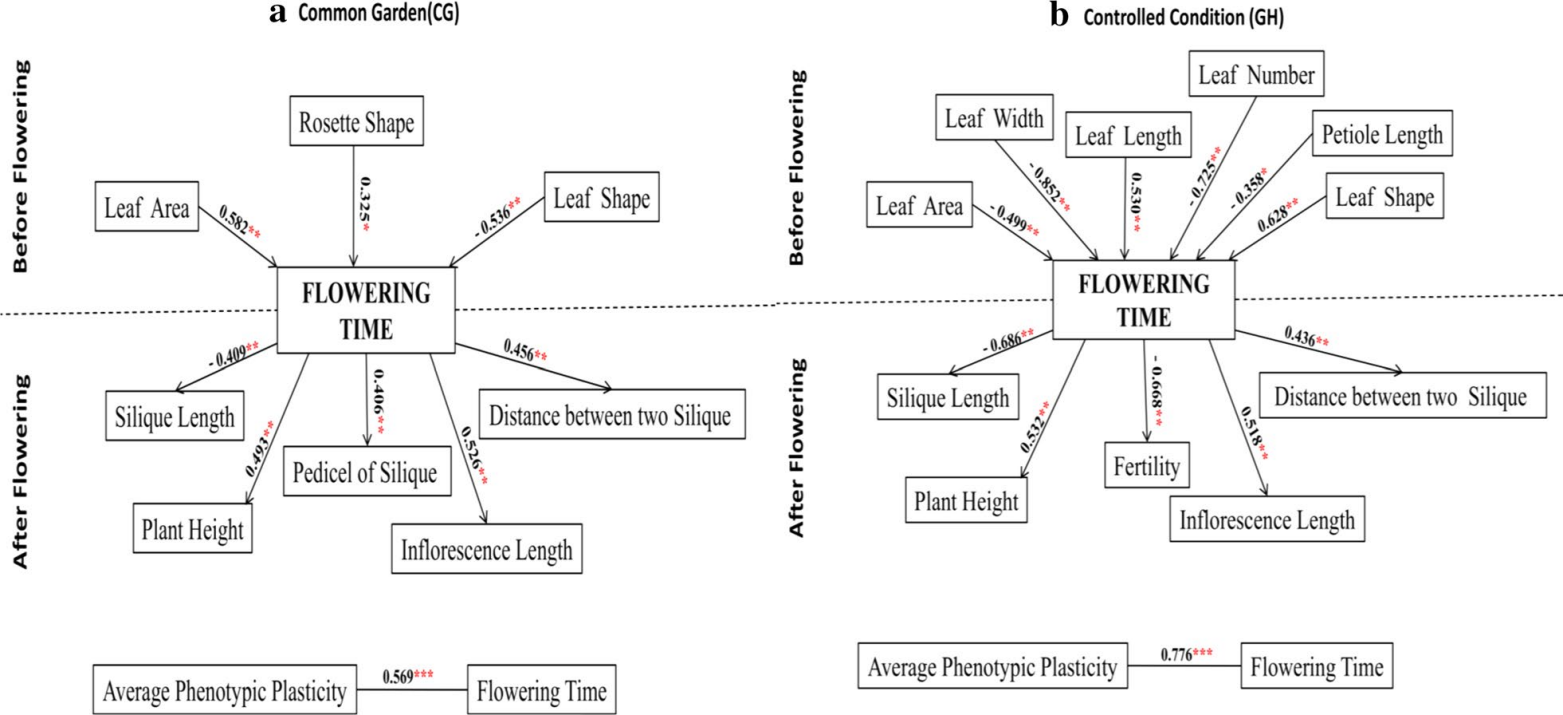
Schematic diagram representing correlation of flowering time with other phenotypic traits: **a** in CG; **b** in GH. The number above the lines shows the correlation coefficient. *P < 0.05, **P < 0.01, ***P < 0.001, *ns* not significant

Correlation of both vegetative and reproductive traits with flowering time within the two conditions also differed both in strength and direction (Fig. 3). In the CG, lower leaf area, rounded rosette, elongated leaf larger siliques with smaller pedicels, lesser distance between two siliques, lesser plant height and inflorescence length were correlated with early flowering (Fig. 3a; Additional file 1: Table S3). While in the GH, flowering time was negatively correlated with leaf number, area and width, petiole length, silique length and fertility percentage and positively with leaf length, leaf shape, plant height, inflorescence length and distance between two siliques (Fig. 3b; Additional file 1: Table S3).

### Phenotypic plasticity of the populations

The P × E interaction term was significant for all the traits indicating for the significant variation in the phenotypic plasticity. In addition to using P × E interaction as a measurement of phenotypic plasticity, we also quantified the amount of plasticity. Significance of variation in the amount of phenotypic plasticity of each trait and population was tested by ANOVA (Additional file 1: Table S4). Interestingly, we found that the variation in phenotypic plasticity between the extreme populations (Chit and Deh) were significant for all the traits. This differentiation decreased as the difference in altitude between the populations decreased. This is because there was no significant variation in the amount of plasticity for leaf shape, number of fruits, number of flowers and flowering time between Deh (lower altitude) and Mun (middle altitude). Although there was variations in slope of reaction norms for the number of fruits and flowers between Deh and Mun but there was no significant variation in their strength i.e., amount of plasticity. The plasticity of all the traits between Mun and Chit (middle and higher altitude) was significant, except plant height (Additional file 1: Table S5). Further most of the reproductive traits of Chit were less plastic as compared to Deh and Mun. On the other hand Mun exhibited greater plasticity for most of the reproductive traits as compared to Deh and Chit (Additional file 1: Table S6). A scatter plot representing the measure of plasticity as residual variation from the best fit line also indicated the same (Additional file 2: Figure S1). The overall plasticity of Chit [0.291 ± 0.038 (mean ± SD)] was significantly higher than Mun (0.262 ± 0.012) followed by Deh (0.181 ± 0.014). The overall phenotypic plasticity was positively correlated with the flowering time in both the conditions (Fig. 3). A clear differentiation of the populations on the basis of the expression of the plasticity of the traits was observed (Fig. 2b).

### Reproductive fitness and path analysis for fitness in the two growth conditions

The expression of the reproductive component of fitness differences between the populations depended on the growth conditions as indicated by significant ‘P × E’ interaction term (F_2,84_ = 9.301; P < 0.0001). Pair- wise comparison of the same population grown under two conditions revealed that the expression of reproductive component of fitness in Deh decreased significantly whereas it increased in Mun and Chit in CG (though not significant). Further, no significant differences in reproductive component of fitness between the populations was found in both the conditions after adjusting for phenotypes and their plasticity (CG − F_2,10_ = 0.802, P = 0.475; GH − F_2,10_ = 1.569, P = 0.255).

**Fig. 4:**
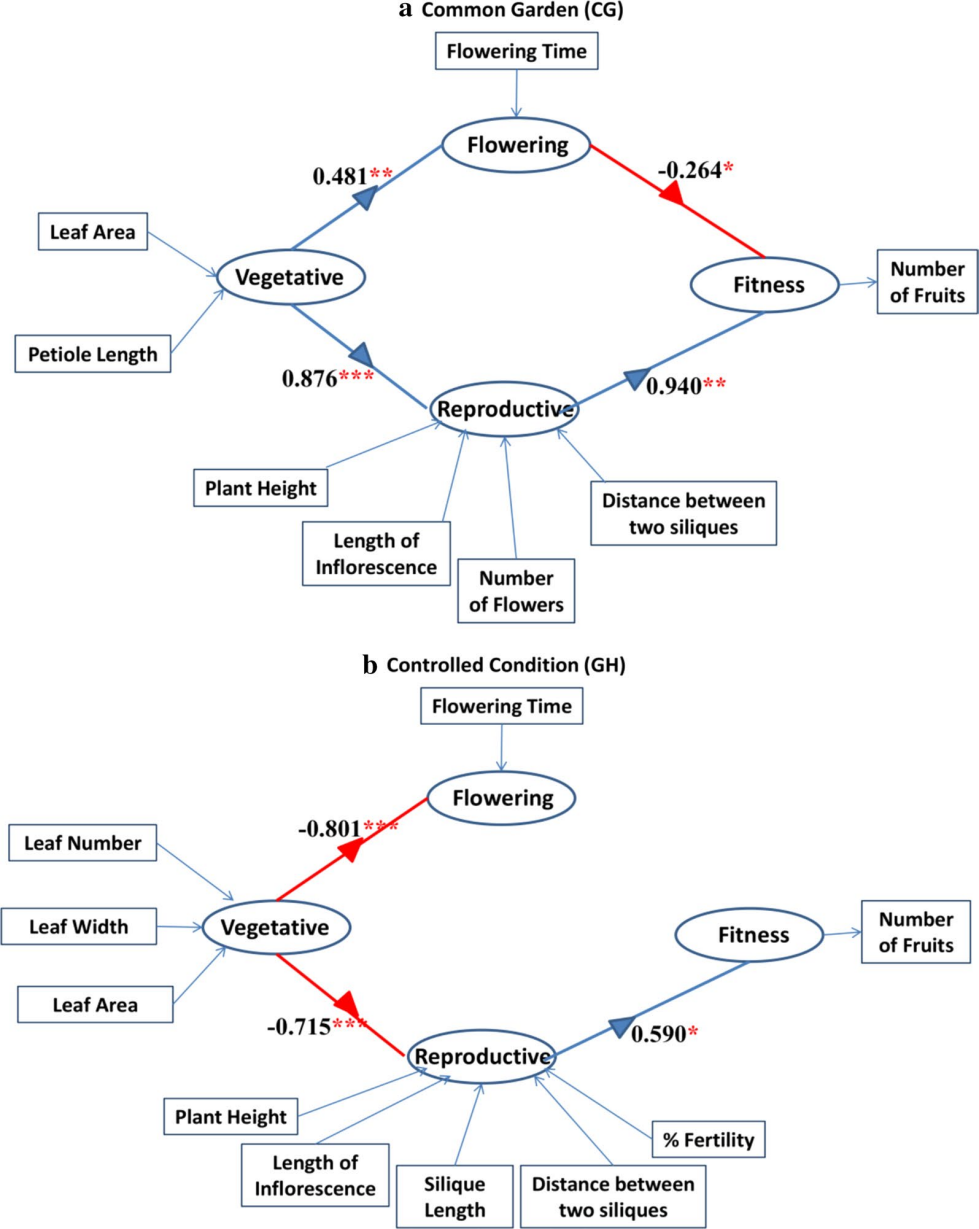
Solved path analysis model showing the influence of vegetative, flowering and reproductive traits on plant reproductive fitness under two conditions. Only significant paths with P < 0.05 are shown. Oval circles represent the latent variables. Blue line shows positive effect and red line shows negative effect. The numbers above lines are path coefficients. Significantly loaded (loading score > 0.7) measured variables are shown in square boxes (*P < 0.05; **P < 0.01; ***P < 0.001). **a** Common garden (CG), **b** controlled condition (GH)

A partial least square path analysis was performed to determine the traits those affect the reproductive component of fitness. The traits contributing to the fitness varied both in strength and direction in the two conditions (Fig. 4; Additional file 1: Table S7). In the CG, the vegetative traits showed a direct positive effect on flowering time and reproductive traits. In contrast, vegetative traits were negatively affecting both these traits in the GH. Reproductive fitness was affected positively by vegetative traits in the CG and negatively in GH. On the other hand, the flowering time negatively affected fitness in the CG. The reproductive traits contributed positively to the fitness in the two conditions but with different strengths. The loading of the individual traits on the vegetative, flowering and reproductive components also varied (Additional file 1: Table S8). Among vegetative traits, petiole length and leaf area were significantly loaded on vegetative component in the CG. In contrast, leaf number, leaf width and leaf area was significantly loaded in the GH. In reproductive component, plant height, length of inflorescence, distance between two siliques, and number of flowers were loaded in CG. While in the GH, plant height, length of inflorescence, size of silique, distance between two siliques and fitness percentage was significantly loaded on reproductive component.

### Broad sense heritability (H^2^) and Qst–Fst comparison

The values of heritability were generally higher for the GH grown plants than the CG [GH = 0.151025 ± 0.040659 (mean ± SE); CG = 0.033834 ± 0.012189 (mean ± SE)]. The mean heritability was generally lower for vegetative traits than reproductive traits in both the conditions (Table 3). The lowest mean heritability was shown by petiole length (mean ± SE = 0.000779 ± 0.000779) under CG condition. In contrast, the highest mean heritability was observed for flowering time under GH condition (0.645 ± 0.204093). At population level, Deh showed the highest mean heritability (0.052575 ± 0.020291) followed by Mun (0.034178 ± 0.02102) and Chit (0.014749 ± 0.008268) under CG condition. In the GH condition Mun (0.071429 ± 0.02444) showed the lowest and Deh (0.213917 ± 0.073223) showed the highest mean heritability.

**Table 3:** Broad sense heritability (H^2^) values for all traits measured in three populations of *A. thaliana* under two conditions

Traits	Common garden (CG)			Controlled condition (GH)		
	Deh	Mun	Chit	Deh	Mun	Chit
Leaf number	0.152 (0.039–0.438)	0.023 (0–0.996)	0	0	0	0
Leaf length	0	0	0	0	0	0.146 (0.036–0.436)
Leaf width	0	0	0	0	0	0.102 (0.013–0.488)
Leaf area	0	0	0.069 (0.003–0.627)	0	0	0.193 (0.070–0.433)
Leaf shape	0	0	0	0	0	0
Petiole length	0.002 (0–1)	0	0	0.130 (0.018–0.549)	0.220 (0.086–0.459)	0.121 (0.019–0.493)
Rosette area	0	0	0.063 (0.002–0.666)	0	0	0.072 (0.003–0.652)
Rosette shape	0	0	0	0.019 (0–1)	0	0.036 (0–0.930)
Flowering time	0	0.043 (0–0.964)	0	0.815 (0.763–0.857)	0.241 (0.109–0.453)	0.886 (0.872–0.899)
Plant height	0	0	0	0.824 (0.770–0.867)	0	0
Length of inflorescence	0	0.005 (0–1)	0	0.750 (0.678–0.810)	0.008 (0–1)	0
Silique length	0.277 (0.133–0.489)	0.192 (0.049–0.520)	0	0.242 (0.110–0.451)	0.073 (0.004–0.600)	0.668 (0.587–0.739)
Pedicel of silique	0.128 (0.021–0.497)	0.318 (0.168–0.517)	0	0.067 (0.003–0.637)	0.038 (0–0.920)	0.274 (0.124–0.501)
Distance between two siliques	0.069 (0.001–0.830)	0	0	0.431 (0.287–0.588)	0.184 (0.063–0.432)	0.258 (0.083–0.574)
Number of flowers	0.170 (0.042–0.491)	0	0.002 (0–1)	0.207 (0.069–0.479)	0.209 (0.046–0.590)	0
Number of fruits	0.096 (0.006–0.643)	0	0.116 (0.013–0.572)	0.152 (0.031–0.503)	0.241 (0.066–0.589)	0
Fertility percentage	0	0	0	0	0	0.090 (0–0.973)

The values in brackets represent the 95% confidence interval

**Fig. 5:**
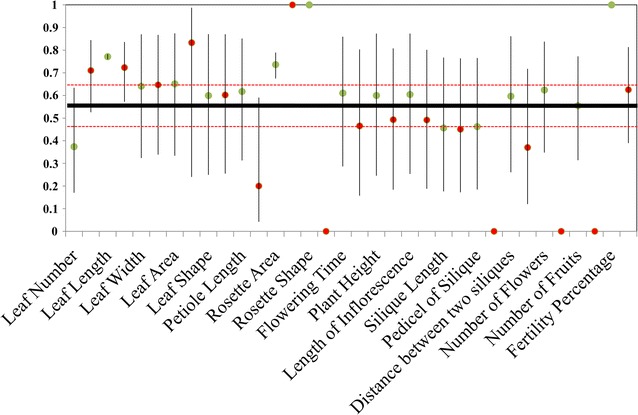
Q_ST_ estimates with 95% confidence interval for 17 morphological traits of three populations measured in two conditions. Green circles for common garden (CG) and red circles for controlled condition (GH). Full line (black) represents the F_ST_ value with its 95% confidence interval (dashed red lines)

Previously, we reported the genetic differentiation as F_ST_ for these populations [23]. The estimated F_ST_ for the three populations was 0.543 (95% CI = 0.44–0.63). Overall the total variance of traits explained as genetic component (Q_ST_) was higher in the CG than GH [(mean ± SE) CG = 0.6411 ± 0.04; GH = 0.447 ± 0.075]. The Q_ST_ estimated ranged from 0 to 1 for GH grown plants and from 0.37 to 1 for CG grown plants representing the level of differential quantitative differentiation (Fig. 5). Q_ST_ was found to be significantly higher than F_ST_ for leaf length, rosette area, rosette shape and fertility percentage under the CG condition. Under GH condition, Q_ST_ of only rosette area was higher than F_ST_ representing complete differentiation and divergent selection. Few traits like rosette shape, pedicel of silique, number of flowers and fruits did not varied at population level in the GH condition. For the rest of the traits, the confidence interval of Q_ST_ and F_ST_ overlapped, suggesting for neutral differentiation.

### Effect of altitude of origin of populations in the two conditions

The phenotypic variation observed in both the growth conditions was significantly associated with the altitude of origin of the populations (CG − Wilk’s lambda = 0.0001; *F* = 139.9; *P* = < 0.0001; GH − Wilk’s lambda = 0.002; *F* = 34.9; *P* = < 0.0001). 99% of the total multivariate trait variation co-varied with the altitude as described by the canonical correlation analysis for both the sites (CG-R_c_^2^ = 0.99, Wilk’s statistic = 0.005; GH-R_c_^2^ = 0.99, Wilk’s statistic = 0.020). The variation in the expression of the phenotypic plasticity by the three populations was also significantly associated with the altitude of origin (Wilk’s lambda = 0.0001; *F* = 712.7; *P* = < 0.0001; R_c_^2^ = 0.99, Wilk’s statistic = 0.002).

## Discussion

Phenotypic trait expression is the effect of interaction between genotype and environment. Populations that grow under different climatic conditions are good source for studying these interactions and their effect on phenotype. In this study, we determined the phenotypic differentiation of natural populations of *A. thaliana* originating from different altitudes. These Indian populations are genetically diverse varying significantly from other world populations and are characterised by strong population structure [23]. The populations showed a clear differentiation on the basis of both absolute phenotype and their plasticity as demonstrated by the discriminant analysis. The existing genetic variations in these populations might be responsible for observed phenotypic variation of traits. The phenotypic variation can be achieved either in the form of adaptive differentiation (for traits that show similar trends in the phenotypic expression in the two conditions) or random phenotypic variation (for traits that show different trends in phenotype in the two conditions). For example, in Deh higher number of leaves and longer siliques were observed in both the conditions. In our earlier study, involving the same but native populations also showed that Deh had higher leaf numbers and longer siliques [27]. This indicates that these traits are fixed. On the other hand, leaf and rosette shape were variable (varying non-significantly in CG and significantly in GH) when compared between CG and GH. Again, these two traits were not variable in their native sites [27]. Differences in the level of significance among the populations and the environmental conditions indicate that these characters are merely random in response and lack any adaptive differentiation. These populations, thus show both an adaptive differentiation and random response depending on environmental condition.

Among the vegetative traits, leaf size and numbers are important adaptive traits. These traits in turns are correlated with other associated rosette and leaf traits. Larger and greater number of leaves was observed in the CG than the GH. Size variation have a direct effect on whole-plant growth rate, mainly through changes in the conductance of the boundary layer, affecting exchange of heat, uptake of carbon dioxide and transpiration [28]. This is mainly due to the fact that larger leaves having thicker boundary layer of slower convective heat loss tends to be hotter than ambient air temperature [29]. In CG there was significant difference in day and night temperature. The large leaf in CG might help to maintain hotter temperature than the ambient air temperature prevalent during night [29]. However, this might be a limitation during water deficit conditions, but we provided sufficient water throughout the growth period. Thus, though edaphic factors and day temperature in field (~ 22–23 °C) were similar with that of GH, there was significant difference in the light intensity and day/night temperature difference between the two conditions, responsible for above observation.

Besides vegetative growth, flowering time is an important indicator of plant fitness. We observed significant differences in the flowering time between the two conditions as well as among the populations in a particular condition. Flowering time of Deh and Mun increased in the CG condition. This was in accordance to the earlier study that showed an increase in flowering time in the field conditions [16]. In contrast, we observed a decrease in flowering time in the field conditions as compared to GH for higher altitude population (Chit). This may be due to the fact that while delayed bolting under benign conditions can lead to higher fecundity [30], early flowering is advantageous in habitats where mortality is likely to be early [31–34]. The winter (Dec–Jan) and spring (Feb–March) seasons in Lucknow, India is followed by hot and dry summer, where temperature rises beyond 40 °C which is unfavourable for growth of *A. thaliana* as against more suitable benign condition in GH. Thus, the CG grown Chit plants completed their life cycle earlier before the onset of unfavourable condition. On the other hand, the climatic factors in field condition of earlier study [16] was more favourable where the maximum temperature recorded during experiments was 28 °C. Further, this trend of flowering time indicates population specific response to climatic condition in context of flowering time. The high altitude population (Chit) might flower early, sensing the unfavourable condition in the CG whereas in GH it has favourable condition to prolong the life cycle. These findings indicate population specific dependency in flowering time under different conditions. This variation in flowering time among the populations is commonly reported in *A. thaliana* [16, 35, 36].

The reproductive traits (measured at the end of life cycle), represents the overall productivity, reproductive allocation and development of the plants. Among other reproductive traits, plant height and length of inflorescence are important traits. These traits were also correlated either positively or negatively with the most of the other traits. A number of studies describing shade avoidance syndrome have also shown an increase in plant height under shade conditions (e.g. [25, 37, 38]). It has also been suggested that low light intensity favours the internodal elongation and hence an increase in plant height [39]. The increase in plant height observed in GH grown plants might be due to the low light in GH as compared to the CG. Seed sizes and germination ability, a measure of maternal investment also determine the plants adaptability under a particular environment [40, 41]. Though there was no significant differences in these traits between any pair of populations in either conditions but when compared between the two environments, seed size (weight) increased in all the populations in CG. More particularly, the seed size differences were more in higher altitude populations (higher plasticity). This may be due to the higher adaptability of high altitude plants in more heterogeneous climatic conditions that is prevalent at high altitude as compared to low altitude plants. High altitude plants may produce more of these reproductive traits for better performance under such heterogeneous conditions.

The observed significance of variation in phenotypic plasticity of the populations implies an underlying genetic polymorphism commonly found in *A. thaliana* [42, 43]. However, the greater overall plasticity observed in the highest altitude plant (Chit) indicates that being exposed to more heterogeneous and stressful environment might have selected individuals having greater plasticity. The greater plasticity in the high altitude population in turn helps them to respond to more stressful and heterogeneous condition of CG than GH. The Deh population experiencing less heterogeneous and stressful conditions suffered a comparative loss of reproductive fitness in the CG. This was in accordance with the earlier study where gene ontology term enrichment of SNP containing genes for the lowest altitude population (Deh) did not retrieved any abiotic stress related term, while high altitude population (Mun and Chit) showed enrichment of GO terms related to high altitude stresses [24]. Plasticity of the traits increases the ecological tolerance [44, 45] which results in higher fitness. Thus the differences in the reproductive fitness observed between the populations were nullified when all the measured traits and their plasticity were included. Selection of more plastic genotypes for environmental heterogeneity has also been reported in other studies [46, 47].

Further, partial least square method of path analysis showed that the different traits were differentially regulating reproductive fitness in the two conditions. The flowering time has been shown to significantly affect fitness of plants [4, 16, 35, 48]. But this association was observed in the CG only. The adverse effect of increase in flowering time on the fitness was in accordance with earlier studies [48–50]. The vegetative traits were shown to affect reproductive fitness through its correlated effects on flowering time and reproductive traits.

The broad sense heritability of the traits differed between the two growth conditions. This observation for heritability of traits in two conditions suggests that heritability was a function of both genotype and environment. Overall the total variance of traits explained as genetic component (Q_ST_) was higher in the CG than GH. This also suggests that the expression of genetically driven phenotypes depends on the environmental condition. The Q_ST_–F_ST_ comparison of these traits helps in predicting the role of neutral or selective divergence in shaping phenotypic variation. Although the method of estimation has been criticized for its biasness due to its inherent theoretical caveats, but still it is commonly used to identify the adaptive traits [51, 52]. Based on this approach we found that the different traits were under divergent selection in the two conditions. The only trait that was under selection in both the condition was rosette area, predicting it to be relevant under two conditions. Interestingly, the confidence interval of Q_ST_ and F_ST_ of flowering time overlapped suggesting its neutral divergence in these populations. In other studies this trait has been shown to be under local divergent selection both in the field and controlled conditions [16, 53]. The significance of variation of this trait in the two conditions and neutrality of its selection suggests for genetic drift effect. However, one needs to be careful in interpreting the data as accession used for estimating Q_ST_ and F_ST_ were different but from the same populations.

Further, there was a significant multivariate association of all the phenotypic traits and their plasticity with the altitude of their native sites. This association of native altitude with the traits that were measured in the uncorrelated environmental conditions (CG and GH) predicts for an altitudinal divergence commonly found by other studies [17, 35, 37, 54, 55]. The similar association of altitude and climate with phenotypic and genetic data were observed earlier using same set of populations [23, 24, 27]. However, our analysis is limited due to use of low number of populations and thus caution may be taken to interpret this particular result.

## Conclusions

West Himalayan populations of *A. thaliana* showed significantly variable morphological response when grown in two different conditions. The traits that could be important for fitness under field condition may not play significant role under controlled conditions. These populations also showed differential genotype–environment interaction for all the traits studied. Further, the high altitude population was more plastic and had higher reproductive fitness in CG as compared to the low altitude populations. The high altitude populations of Himalaya are exposed to harsh environmental condition and thus might have evolved to fit well in the heterogeneous condition (like CG), hence showing maximum plasticity and reproductive fitness as compared to the lower altitude populations. However, experimental validation needs to be performed using reciprocal transplant experiment to prove the observed phenomenon. We also emphasise that caution may be taken to interpret these results due to small population size. However, more efforts are being made to phenotype more populations from this region.

## Methods

### Study population and experimental design

**Fig. 6:**
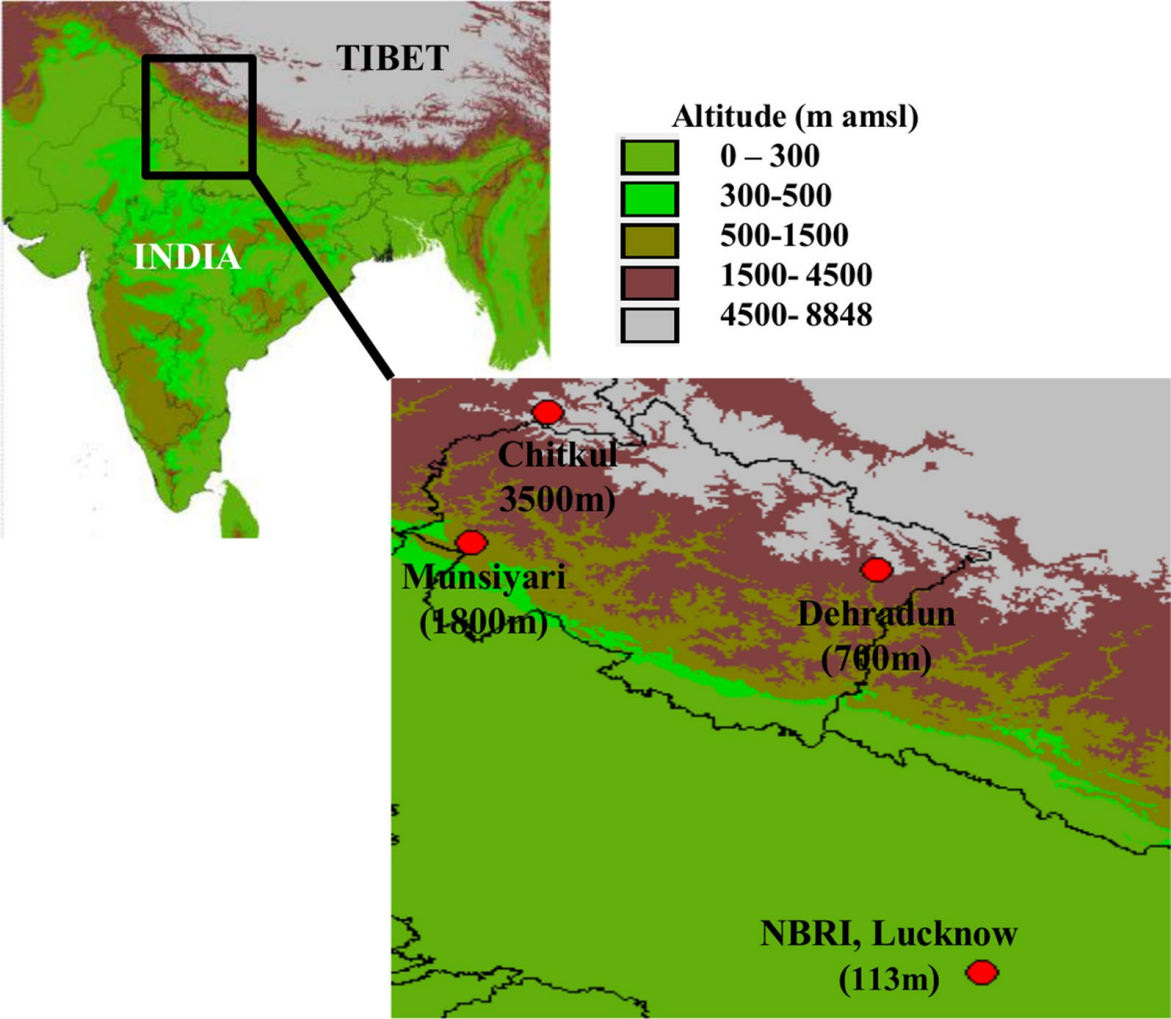
Map of India showing the sites of collection of population along with the experimental sites. The map was downloaded from and created by open source software DIVA-GIS (http://www.diva-gis.org/Data)

The details of the populations and climatic condition of the native sites have been reported earlier [27]. Seeds were collected from 15 accessions from each of the three sites [viz., Deh (700 m above msl); Mun (1800 m above msl); and Chit (3400 m above msl)] maintaining a minimum distance of 2 m between the two accessions. Seeds were grown for one generation under controlled conditions in order to eliminate effect due to level of seed maturity in field conditions. Stratified seeds were grown under two environmental conditions: (1) controlled conditions (GH) with 16 h/8 h light/dark; temperature 20–22 °C, and light intensity—120 PPFD; (2) common garden (CG) at Council of Scientific and Industrial Research-National Botanical Research Institute (CSIR-NBRI), Lucknow, India (details in Additional file 1: Table S9, Additional file 2: Figure S2). To check the best environmental condition for germination of seeds and growth of the plants in CG, batches of seed were sown at weekly interval starting from mid November to mid December in the previous year. The appropriate environmental condition for the growth of *A. thaliana* in CG was found to be from the last week of November to March. Subsequently, experiment was set up during last week of November, 2013 in randomized-block factorial design consisting of two factors: population (P) and growth condition (E) viz. CG and GH. 180 pots (4 replicate × 15 accessions × 3 populations) filled with Soilrite Mix^®^ (Keltech Energies Ltd. Perlite Division, India.) were planted with five seeds per pot for each environmental condition. After germination, only one healthy seedling per pot was maintained. The plants were watered regularly. The accessions were randomised within the trays per block to avoid positional effects. The CG, GH and native sites of the populations is shown in Fig. 6.

### Phenotypic trait and plasticity measurement

A total of 17 vegetative, flowering and reproductive traits were measured during the experiment. Vegetative traits were measured after 5 weeks of germination while reproductive traits were measured after completion of life cycle (Additional file 1: Tables S10, S11). Leaf area was estimated as product of length and width, while leaf shape was represented as a ratio of length to width. Rosette area was measured as product of major and minor axis and rosette shape as a ratio of major to minor axis. We considered both the leaf and rosette shape as rounded when the ratio is close to one and elongated if the ratio was more than one. The plants were censused in every two days for the estimation of flowering time until all plants have bolted. Flowering time was estimated as number of days from germination to bolting. After the completion of life cycle, percentage of total number of flowers produced that converted into fruits was used to estimate fertility percentage. Being highly correlated with seed production, total reproductive fitness was measured as the number of fruits produced [16, 56]. The seed weight was estimated by measuring the weight of 100 seeds. This measurement was taken randomly for six accessions from each population and for each condition (6 accessions × 3 population × 2 environment). For each condition, equal number of seeds from each of the 15 accessions per population was pooled in a replicate of four (3 population × 2 environment × 4 replicate) for the estimation of germination percentage after 5 days.

Amount of phenotypic plasticity was calculated for each trait as the change in mean trait value in one environment to the other [57]. Least square means of each accession was used for calculating phenotypic plasticity. Overall plasticity of each accession was estimated as the average of plasticity of each trait. A bootstrapping procedure, with 1000 iterations was used to obtain confidence interval and error estimate for the plasticity, using *bootMer* function of *lme4* package in R [58]. The error estimate for overall plasticity was calculated as average of the errors obtained for all traits. A scatter plot of raw trait values between the two conditions was made to visualize the phenotypic plasticity as residual deviation from the best fit and expected line (observed–expected).

### Statistical data analysis

All the statistical analysis (except mixed model ANOVA) was performed with statistical software IBM-SPSS 22 (IBM Corp., released 2013, IBM SPSS Statistics for windows, Version 22.0. IBM Corp, Armonk, NY). Leaf shape was square root transformed to meet the assumption of normal distribution. All the analysis was performed on least square mean of replicates estimated for each accession per population. In our previous study, we have shown that these populations harbour considerable amount of genetic variation within population [23]. Thus, each accession was considered as replicate within population for statistical analysis. ANOVA was performed to find the trait differentiations between the populations in the two conditions separately. Post hoc bonferroni test was used to compare the population pair wise.

A mixed model ANOVA was conducted to identify the differences in the expression of phenotypic traits with the interaction of the environment by nlme package of R [59]. ‘Population (P)’, ‘environment (E)’ and ‘population × environment (P × E)’ interaction were used as fixed factors and accessions as random factor. Replicates were considered as blocks. Discriminant analysis was performed to aid the visualization of population differentiation. The significance of variation in the amount of plasticity was also analysed using one way ANOVA and visualized via discriminant analysis.

Correlation among the populations for different traits within and between the two conditions was analysed using Pearson’s correlation. A correlation of all the phenotypic traits with the flowering time was also calculated to predict whether the difference in the flowering time was related to the phenotypic traits and their plasticity.

To test the differences in performance of the three populations in the two sites, ANOVA, for relative and absolute reproductive fitness was conducted with population (P) and growth condition (E) as fixed effects and accessions as random effect. A significant ‘P × E’ term for relative reproductive fitness was used as indication of fitness differences present between the populations that depends on the environment. To find out the extent to which environment dependent selection on plastic characters explains these fitness differences between the populations, ANCOVA was conducted on relative reproductive fitness as dependent variable and all the measured traits and their plasticity as covariate. Further in order to study potential interrelationship and effect of vegetative, flowering and reproductive traits on the reproductive fitness of the plants in the two conditions, partial least square regression path analysis was performed. We utilized partial least square method of path analysis. This method is different from conventional covariance-based method as it do not impose any assumption on data distribution [60]. The traits were divided into vegetative, flowering and reproductive variables to predict fitness as number of fruits produced. All these traits are supposed to influence fitness of plants. The hypothesized model for path analysis is shows in (Additional file 2: Figure S3). Loading of traits greater than 0.7 on their respective variables were considered to be significant. Confidence intervals of the path coefficients were calculated using 1000 bootstraps. This analysis was performed in *plspm* package of R [61].

Broad sense heritability was estimated for each population and each trait as H^2^ = V_G_/(V_G_ + V_E_), where V_G_ is among accession variance and V_E_ is residual variance [16, 53]. The amount of quantitative genetic variation, Q_ST_ [62] was estimated for all the traits. Q_ST_ was estimated assuming complete selfing as V_B_/(V_B_ + V_W_), where V_B_ is among population variation and V_W_ is within population variation [16, 53, 63]. 95% confidence intervals were calculated for both Q_ST_ and H^2^ using restricted maximum likelihood (REML) variance components [64]. F_ST_ used as a measure of genetic differentiation was calculated from previously published microsatellite data of these populations [23]. The comparison of Q_ST_ and F_ST_ is commonly used method to differentiate selection from neutral divergence for the traits. Q_ST_ > F_ST_ suggests for divergent selection, while Q_ST_ < F_ST_ predicts for uniform selection. The traits that show Q_ST_ = F_ST_ are under neutral divergence [65, 66]. Though, this method (Q_ST_–F_ST_ comparison) of identifying selection has been predicted to be biased but is still used to identify traits under selection [66]. The Q_ST_ was considered to differ significantly from F_ST_ only when their confidence intervals did not overlap.

The significance of impact of altitude of native sites of the populations on the expression of traits and their plasticity was assessed using MANOVA. Canonical correlation was also performed to account for percentage of variance in the phenotypic traits explained (using squared canonical correlation coefficient) by altitude of the site of origin. These analyses were performed individually for CG, GH and phenotypic plasticity.

## Additional files

**Additional file 1: Table S1.** Post-hoc (Bonferroni) test (P values) for pair wise comparison of traits between the three populations of *Arabidopsis thaliana* in common garden (CG) and controlled condition (GH). Significant figures are in bold. **Table S2.** Analysis of significance of effect of population and growth environment (CG vs. GH) on vegetative, flowering and reproductive traits for three populations of *Arabidopsis thaliana* using mixed model ANOVA. **Table S3.** Correlation between morphological traits measured within the two conditions. The upper triangle shows Pearson correlation (r) between traits within the common garden (CG) and lower triangle for within the controlled condition (GH). Significant values are shown in bold (*P < 0.05; **P < 0.01; ***P < 0.001). **Table S4.** Analysis of variation for amount of phenotypic plasticity. The significance of variation in phenotypic plasticity among the three populations was tested using ANOVA. All traits were significant at P value < 0.05. **Table S5.** Post-hoc (Bonferroni) test (P values) for pair wise comparison of plasticity for all the traits between the three populations of *Arabidopsis thaliana.* **Table S6.** Phenotypic plasticity of the three populations of *Arabidopsis thaliana*. The confidence interval of plasticity was calculated using 1000 iterations. **Table S7.** Coefficients for direct, indirect and bootstrap estimated total effect for the latent variables used for path analysis in the two condition. Total effect is sum of direct and indirect effect. Numbers in brackets represent 95% confidence interval for total effect. **Table S8.** Loading and weight of all the traits on their latent variable (blocks) estimated using partial least square path analysis. 95% confidence intervals estimated using 1000 bootstrap are shown in brackets. **Table S9.** Average monthly weather conditions of Common garden site at Lucknow, India during the growth period. Altitude: 113 m above mean sea level; Geographical coordinates: 260 55′ N, 800 59′ E. **Table S10.** Ls mean of phenotypic traits for all accessions with 95% confidence interval. **Table S11.** Raw data of all the phenotypic traits measured for all accessions under the two growth conditions.

**Additional file 2: Figure S1.** Scatter plot of accession means in CG vs GH. The observed line is shown as dotted red line and best fit line by solid black colour. **Figure S2.** Log climatic conditions of Common garden site at Lucknow, India during the growth period. (a) Daily Average Temperature; (b) Daily Maximum Temperature; (C) Daily Minimum Temperature; (D) Daily Light Intensity. Altitude: 113 m above mean sea level; Geographical coordinates: 260 55′ N, 800 59′ E. The weather data of CG was obtained from Amausi weather station, Lucknow situated at around 16 km from CG Freely available from- http://en.tutiempo.net/climate/ws-423690.html, Accessed 5th October 2015. **Figure S3.** Model used for path analysis. Oval circles represent the latent variables with their measured variables in the square boxes.

## Abbreviations

CG: common garden set up CSIR-National Botanical Research Institute, Lucknow
GH: controlled environmental conditions used for plant growth
P: population factor
E: growth condition (environment factor)
P × E: population and growth condition interaction term (factor)
H^2^: broad sense heritability
Q_ST_: quantitative population differentiation
F_ST_: genetic differentiation
