# Supplementary material for: High altitude population of Arabidopsis thaliana is more plastic and adaptive under common garden than controlled condition
Source: BMC Ecol. 2017 Dec 13;17:39. doi: 10.1186/s12898-017-0149-5 (PMC5729231; doi:10.1186/s12898-017-0149-5)

Additional File 2:

Figure S1: Scatter plot of line means in CG vs GH. The observed line is shown as dotted red line and best fit line by solid black colour.

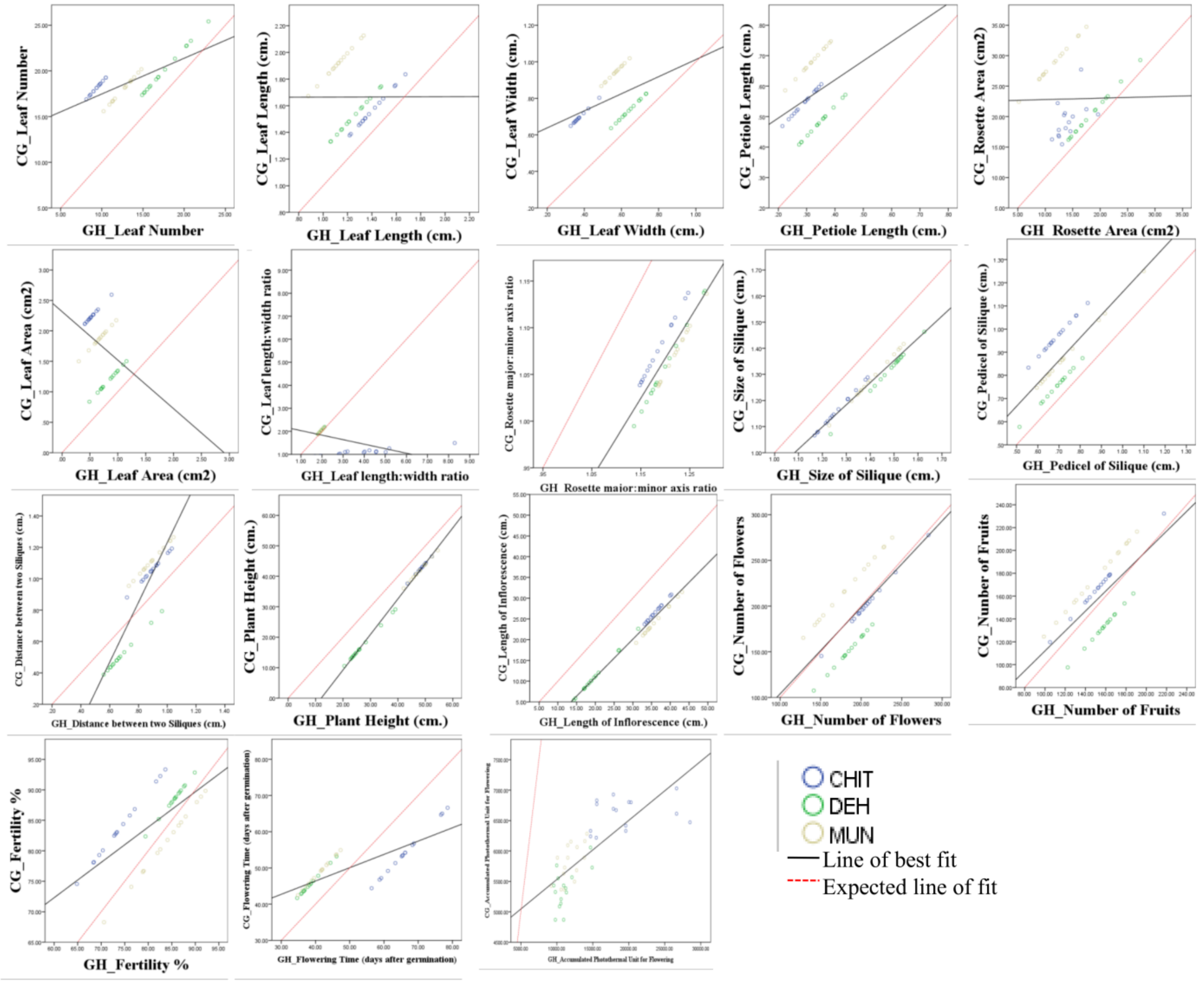

Figure S2: Log climatic conditions of Common garden site at Lucknow, India during the growth period. (a) Daily Average Temperature; (b) Daily Maximum Temperature; (C) Daily Minimum Temperature; (D) Daily Light Intensity. Altitude: 113 m above mean sea level; Geographical coordinates: 260 55’ N, 800 59’ E. The weather data of CG was obtained from Amausi weather station, Lucknow situated at around 16 km from CG Freely available from- http://en.tutiempo.net/climate/ws-423690.html. accessed 5th October 2015.

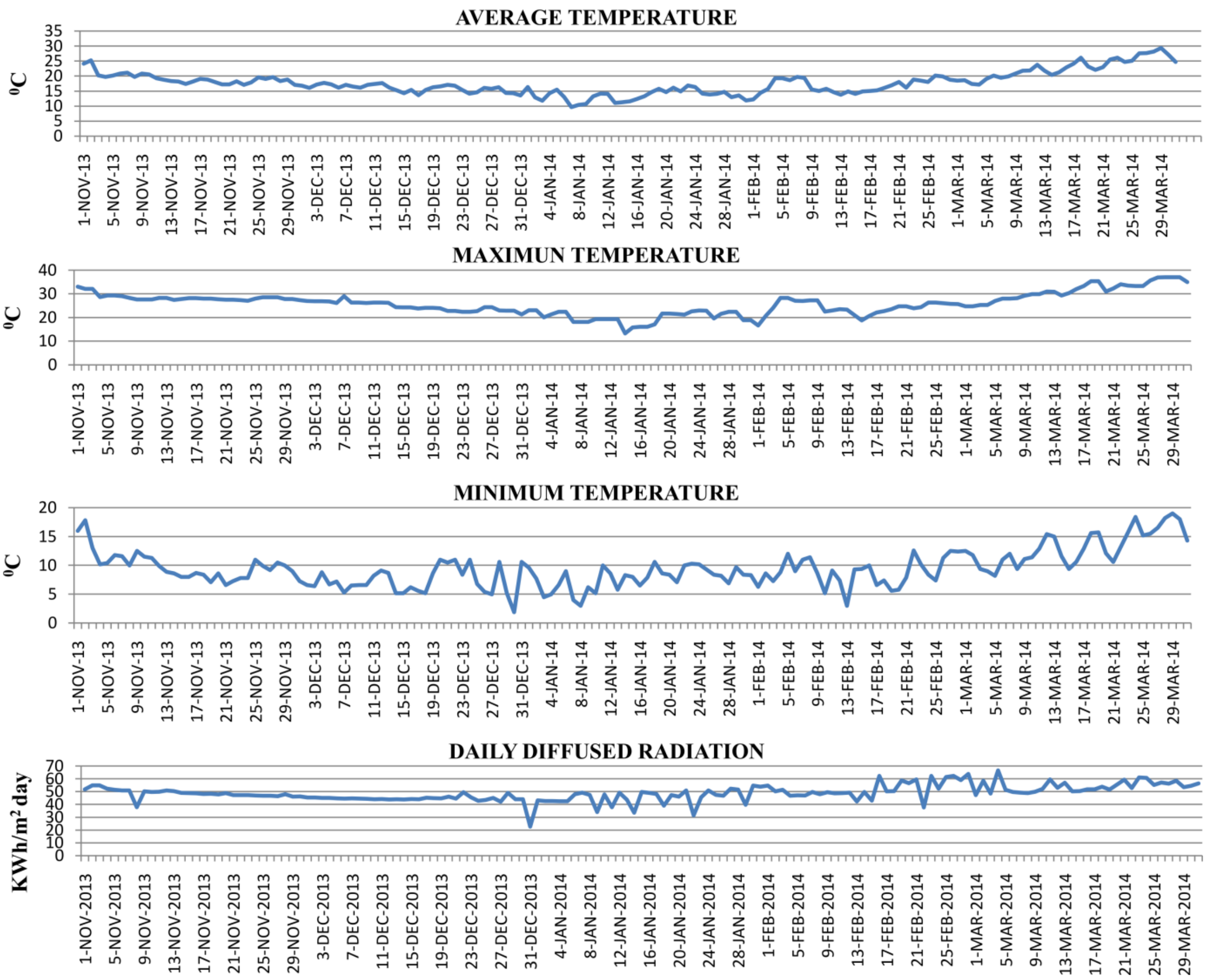

Figure S3: Model used for path analysis. Oval circles represent the latent variables with their measured variables in the square boxes.

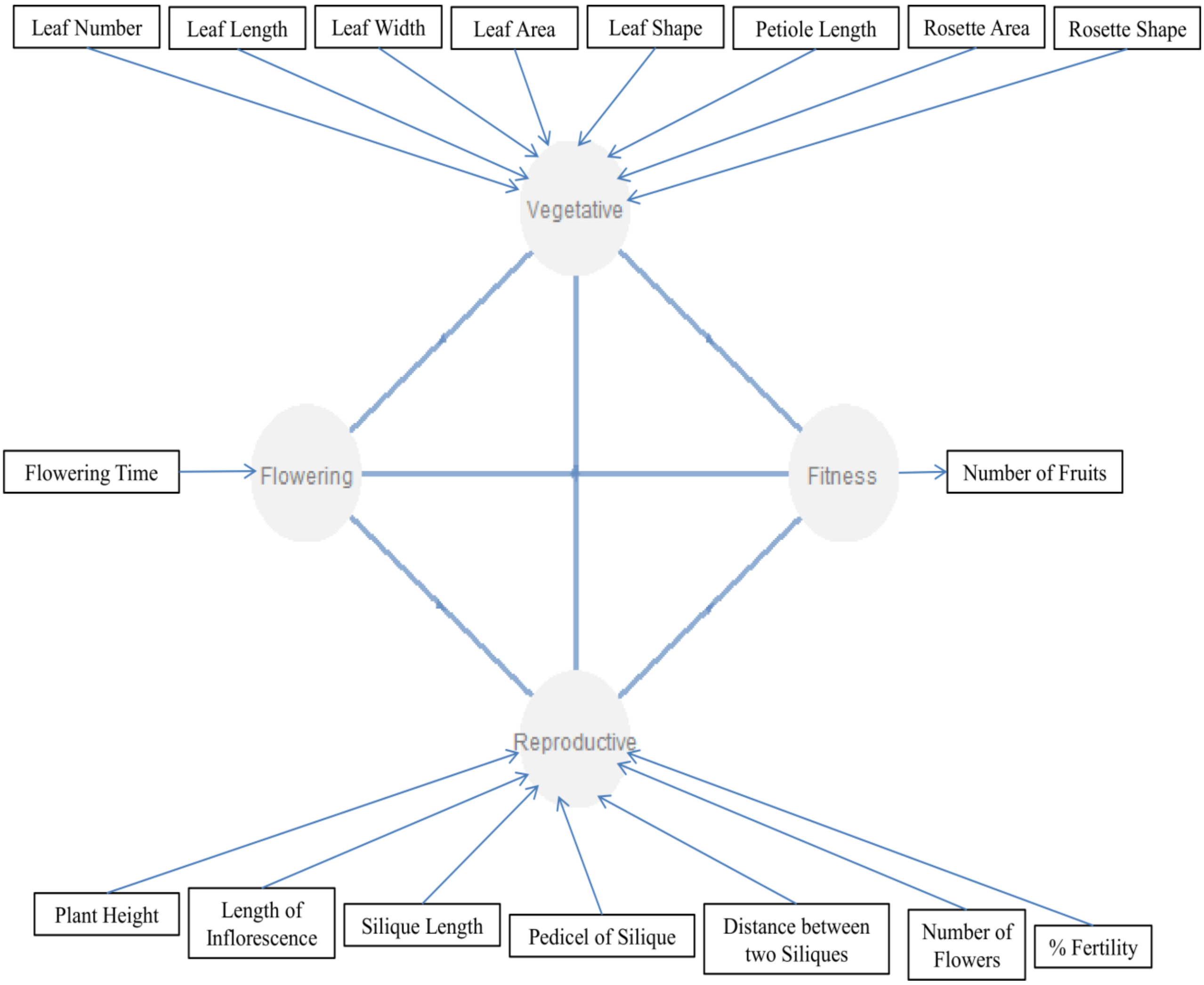

Supplement: Supplementary file 2 — Additional file 2: Figure S1. Scatter plot of accession means in CG vs GH. The observed line is shown as dotted red line and best fit line by solid black colour. Figure S2. Log climatic conditions of Common garden site at Lucknow, India during the growth period. (a) Daily Average Temperature; (b) Daily Maximum Temperature; (C) Daily Minimum Temperature; (D) Daily Light Intensity. Altitude: 113 m above mean sea level; Geographical coordinates: 260 55′ N, 800 59′ E. The weather data of CG was obtained from Amausi weather station, Lucknow situated at around 16 km from CG Freely available from- http://en.tutiempo.net/climate/ws-423690.html, Accessed 5th October 2015. Figure S3. Model used for path analysis. Oval circles represent the latent variables with their measured variables in the square boxes. [file 12898_2017_149_MOESM2_ESM.pdf]
